# A Case of Poisoning with Strychnine Relieved by Chloroform

**Published:** 1853-07

**Authors:** Geo. L. Upshur

**Affiliations:** Surgeon to U. S. Marine Hospital, Norfolk, etc., etc.


*A Case of Poisoning with Strychnine relieved by Chloroform.* By Geo. L. Upshur, A. M., M. D., Surgeon to U. S. Marine Hospital, Norfolk, etc., etc.—On the 30th of March 1853, I was called to see the child of Mr. F., sick with chronic diarrhœa. It was born on the 2d of January, and consequently was not quite three months old. When less than two weeks old, its bowels became disordered, and I had frequently prescribed medicine for it from the description given of the case by the father.

It took first one simple astringent and then another, with now and then an alterative dose of calomel, but nothing seemed to exercise any permanent control over the affection. The child thus became, in a short time, much emaciated and enfeebled, and I was requested to visit it on the 30th of March. Found it feverish, thirsty and restless, with movement of the bowels every hour or two, and scarcely any digestive power left. The milk was passed from the bowels in the same state as it left the stomach, and there were frequent nausea and vomiting. I ordered one of the following powders three times a day:

| | | |
|---|---|---|
| ℞. | Ext. Krameriæ | gr. xv. |
| | Pulv. Dover. | gr. xii. |
| | Pulv. Rhei | gr. vj. |
| | Strychniæ | gr. 1-5th. |

M. Chart. 12.

In the course of two or three days there was a manifest improvement in the condition of the patient; the nausea ceased, the dejections became less frequent and more natural, and the mother's milk seemed to agree as it never had done before. The powders were continued until 24 had been taken, when they were discontinued, and I left the child fairly convalescent.

On the 14th of April the father came to my office to say that the child's bowels had again become disordered, but there was no fever, and it was very sprightly. I ordered a tea-spoonful of infusion of rhubarb with six drops of paregoric to be given three times a day. On the 20th there had been no improvement, and I was requested to visit it. Finding it in nearly the same condition that it was on the 30th of March, I determined to give the strychnia again, and in larger doses. The following is the prescription:

| | | |
|---|---|---|
| ℞. | Pulv. Dover. | gr. xv. |
| | Cretæ Prep. | gr. xxx. |
| | Pulv. Rhei | gr. viii. |
| | Strychniæ | gr. ½. |

M. Chart. 15. S. A powder three times a day.

The child took the first dose at one o'clock; at two the mother discovered that it was becoming very restless—frequently starting, as it were involuntarily, and, now and then, becoming quite rigid. These symptoms gradually increased in violence until 4 o'cloek, when I was sent for, the messenger informing me that the child had strong convulsions. Arriving in a few minutes, I found the patient lying in the lap with almost every muscle in the body acting spasmodically. The spasms were tetanic, with very short intervals between the paroxysms. There was no heat about the head, nor, indeed, any feverish action, and the child's intellect was perfectly clear, as was evinced by the cry of suffering which was uttered between the paroxysms, and the earnest look for relief cast upon every person around it. The spasms resembled more nearly than anything else a violent shuddering, or agitation of the whole frame—the hands being tightly clenched, the body bent backwards, and the limbs immovably rigid.

I immediately ordered a mustard plaster to be applied down the spine, and, while I went for some chloroform, directed equal parts of brandy and water to be freely given. I was absent half an hour, and on my return found the convulsions more violent, if possible, than ever. Fifteen drops of chloroform were sprinkled on a towel, folded in the shape of a funnel, and applied over the patient's mouth and nose. In four minutes she was fully under the influence of the remedy, and snored aloud. The towel was removed, and as soon as consciousness began to return the spasms also began to re-appear, when it was again applied. The child was thus kept fully under the influence of the chloroform for four hours, at the end of which time, there being no tendency to a recurrence of the convulsive movement, the remedy was discontinued, the entire quantity inhaled being f℥ss.

For two days afterwards the child had frequent bloody, slimy stools, accompanied by a good deal of pain and fever; but these symptoms yielded readily to a blister on the abdomen, and nitrate of silver enemata, with laudanum. The child is now in better health than she has been since her birth, being four months old on the 2d inst.

*Remarks.*—This case presents some exceedingly interesting points. The tender age of the patient, the very small dose of strychnia—only 1-30th of a grain—the length of time that the chloroform impression was maintained, and the perfectly satisfactory result of the treatment, are all matters of great interest to the medical man. At first I was disposed to doubt the possibility of so minute a dose of strychnia producing such violent symptoms, and began to think that some mistake had been committed by the apothecary; but, upon inquiry, I learned that the prescription had been put up by one of our most careful apothecaries, whose attention was called at the moment to the necessity of being careful in weighing the strychnia, by an older member of the firm. I do not think it probable that more than half a grain was put in the fifteen powders, so that the child could not have taken more than a thirtieth of a grain. When it is remembered, that one twentieth of a grain had, but a few days before, been given every day for eight days, without any unpleasant effects, it seems almost incredible that a thirtieth of a grain should produce such alarming symptoms.

Is it possible, that the action of strychnia, like that of digitalis, is cumulative? If so, then the convulsions in this case may have resulted from the whole amount of the poison which the child had taken from the 30th of March to the 14th of April, instead of from the simple dose of one thirtieth of a grain. But the best writers on Materia Medica do not consider strychnia to be cumulative in its action—certainly not to the same extent as digitalis—for Taylor in his work on Poisons, says, "when the dose is gradually raised, the system may become habituated to the poison, so as to resist the effects of a very large dose;" and Pereira has seen the dose gradually increased, until one grain and a half was taken three times a day, without dangerous consequences. Royle, however, remarks, "that some cases show a tendency to its becoming cumulative in its action."

I am not disposed to believe, however, that the symptoms in this case were due to the cumulative action of the poison. The medicine was entirely discontinued on the 6th of April, and was not resumed until the 14th, eight days after. The symptoms, too, followed the administration of the powder on the 14th too quickly, and were too marked in their character, to be attributed to any other cause than the powder; so, that after a careful review of all the attending circumstances, and without a desire to give undue weight to any one of them, I am satisfied that the child was poisoned by only the thirtieth of a grain of strychnia, and that the dose would have been fatal but for the chloroform.

This is probably the smallest dose of strychnia that ever produced symptoms which threatened to destroy life. Taylor quotes from the *Brit. Amer. Journ.* for Aug., 1847, the case of Dr. Warner, who was killed in fourteen minutes by taking half a grain; and Dr. Watson reported a case, in the *Med. Gaz.*, for 1845, of a girl, æt. 13, who died from taking three quarters of a grain; but in both these cases the dose was larger, in proportion to the age of the patient, than in the case herein reported.

The magic effect of the chloroform deserves some notice. Its impression was maintained for four hours with the happiest results. In less than five minutes the child became perfectly composed, and soon was in a profound sleep. At every recurrence of the convulsive movement, two or three inspirations were sufficient to produce perfect composure, until the force of the poison had expended itself, when the chloroform was discontinued, and the patient recovered without further serious symptoms.

In conclusion, I may be permitted to say, that I report this case only for what it is worth. As I have said elsewhere in the pages of this Journal, it is very unphilosophical to deduce any general principle in therapeutics from a solitary observation, and, therefore, in giving this case to the public I do not intend to commit the error which is so often committed—that of seeking to establish a permanent law from the treatment of a single case. I merely give the facts, viz: that I administered the thirtieth of a grain of strychnia to a child three months and twelve days old; that, in a short time, the child had all the serious symptoms that are attributed to a poisonous dose of strychnia, and which, to all appearance, would have destroyed life had they continued; and that it was kept under the influence of chloroform for four hours, and recovered without having any return of spasm or other serious symptom. I believe the recovery was due to the chloroform.—*Medical Examiner, Philadelphia.*

